# Supplementary material for: Structural mapping of polyclonal IgG responses to HA after influenza virus vaccination or infection
Source: mBio. 2025 Feb 6;16(3):e02030-24. doi: 10.1128/mbio.02030-24 (PMC11898601; doi:10.1128/mbio.02030-24)
Supplement: Supplemental material — Supplemental figures and tables. [file mbio.02030-24-s0001.docx]

**Supplementary Material**

**
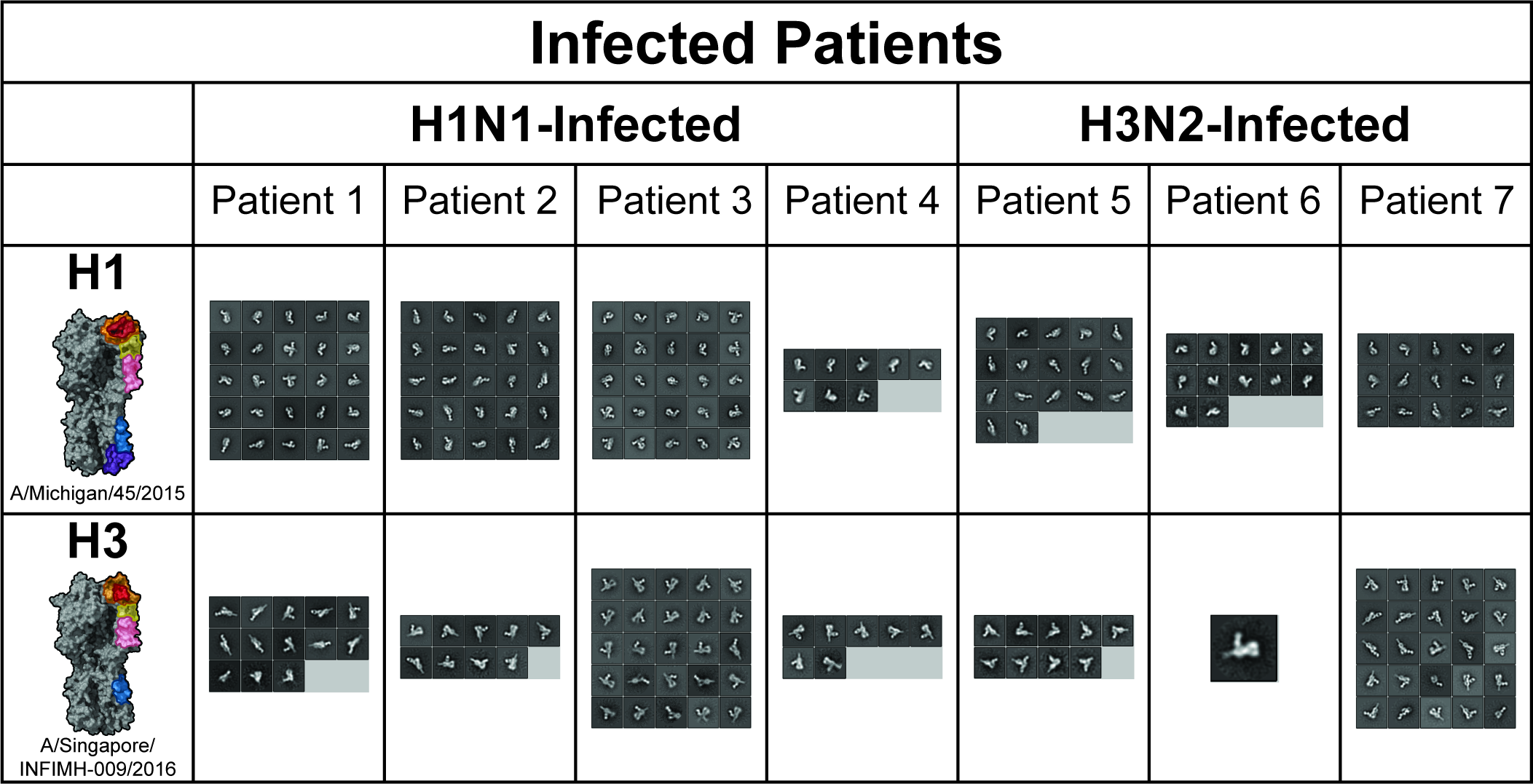
**

Figure S1: Representative 2D classes of infected patient Fabs bound to H1 and H3. Classes are representative of particles incorporated in final 3D reconstructions and/or cartoon Fab representations.

**
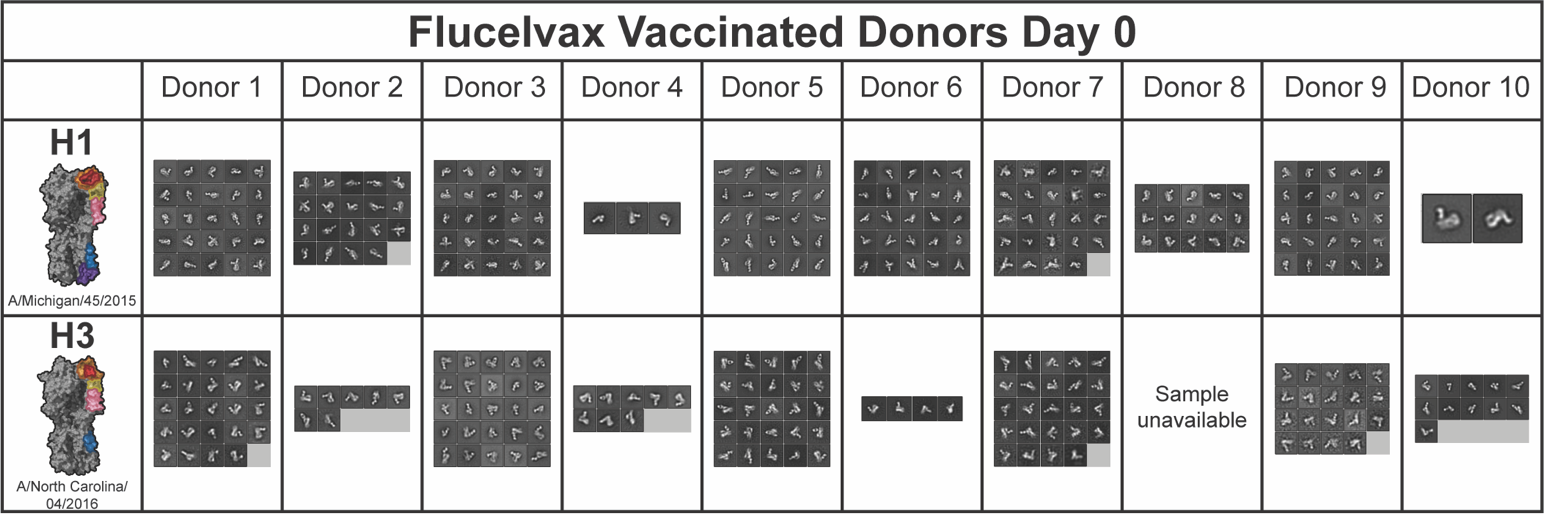
**

Figure S2: Representative 2D classes of vaccinated donor Fabs bound to H1 and H3 at Day 0. Classes are representative of particles incorporated in final 3D reconstructions and/or cartoon Fab representations.

**
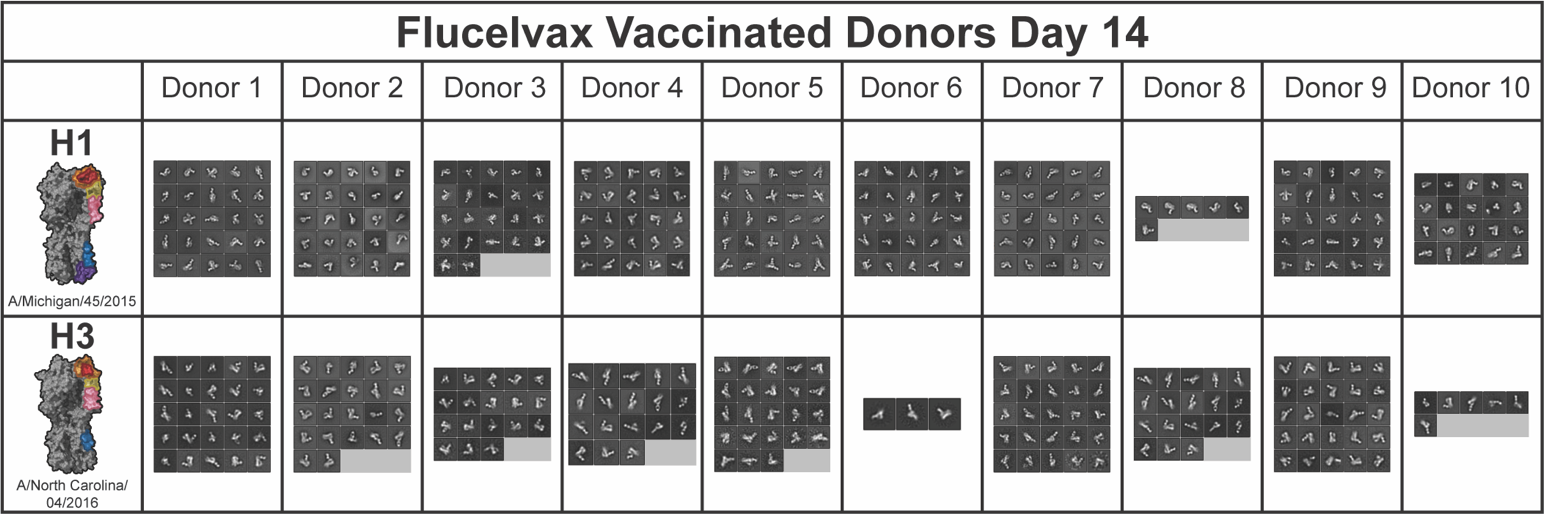
**

Figure S3: Representative 2D classes of vaccinated donor Fabs bound to H1 and H3 at Day 14. Classes are representative of particles incorporated in final 3D reconstructions and/or cartoon Fab representations.

Table S1: Individual microneutralization titers for vaccinated donors. Microneutralization titers against A/Michigan/45/2015 H1N1 and A/Singapore/16-0019-2016 H3N2 from vaccinated donor serum collected at Day 0 and Day 14 reported as reciprocal dilutions.

| Microneutralization A/Michigan/45/2015 H1N1 | | | | | |
| --- | --- | --- | --- | --- | --- |
|  | Donor | Day 0 | Day 14 | Fold change  (Day 14/Day 0) |  |
|  | 1 | 56.57 | 113.14 | 2.0 |  |
|  | 2 | 80 | 113.14 | 1.4 |  |
|  | 3 | 20 | 160 | 8.0 |  |
|  | 4 | 80 | 905.1 | 11.3 |  |
|  | 5 | 320 | 320 | 1.0 |  |
|  | 6 | 640 | 905.1 | 1.4 |  |
|  | 7 | 160 | 160 | 1.0 |  |
|  | 8 | 20 | 20 | 1.0 |  |
|  | 9 | 40 | 80 | 2.0 |  |
|  | 10 | 40 | 56.57 | 1.4 |  |
|  |  |  |  |  |  |
|  |  |  |  |  |  |
| Microneutralization A/Singapore/INFIMH-16-0019/2016 H3N2 | | | | | |
|  | Donor | Day 0 | Day 14 | Fold change  (Day 14/Day 0) |  |
|  | 1 | 40 | 452.55 | 11.3 |  |
|  | 2 | 113.14 | 160 | 1.4 |  |
|  | 3 | 80 | 320 | 4.0 |  |
|  | 4 | 160 | 640 | 4.0 |  |
|  | 5 | 320 | 320 | 1.0 |  |
|  | 6 | 10 | 80 | 8.0 |  |
|  | 7 | 452.55 | 640 | 1.4 |  |
|  | 8 | 640 | 452.55 | 0.7 |  |
|  | 9 | 226.27 | 320 | 1.4 |  |
|  | 10 | 56.57 | 56.57 | 1.0 |  |

Table S2: EMDB deposition ID. Global refinements, half maps, and 3D classes used to generate all negative stain EM reconstructions presented in this manuscript have been deposited to the EMDB and can be accessed using the data provided in the table below.

| **EMDB Title** | **EMDB Accession Code** |
| --- | --- |
| Polyclonal immune complex of Fab binding the H1 HA from serum of patient 1 | EMD-45345 |
| Polyclonal immune complex of Fab binding the H1 HA from serum of patient 2 | EMD-45346 |
| Polyclonal immune complex of Fab binding the H1 HA from serum of patient 3 | EMD-45347 |
| Polyclonal immune complex of Fab binding the H1 HA from serum of patient 4 | EMD-45348 |
| Polyclonal immune complex of Fab binding the H1 HA from serum of patient 5 | EMD-45349 |
| Polyclonal immune complex of Fab binding the H1 HA from serum of patient 6 | EMD-45350 |
| Polyclonal immune complex of Fab binding the H1 HA from serum of patient 7 | EMD-45351 |
| Polyclonal immune complex of Fab binding the H3 HA from serum of patient 1 | EMD-45352 |
| Polyclonal immune complex of Fab binding the H3 HA from serum of patient 2 | EMD-45353 |
| Polyclonal immune complex of Fab binding the H3 HA from serum of patient 3 | EMD-45354 |
| Polyclonal immune complex of Fab binding the H3 HA from serum of patient 4 | EMD-45355 |
| Polyclonal immune complex of Fab binding the H3 HA from serum of patient 5 | EMD-45356 |
| Polyclonal immune complex of Fab binding the H3 HA from serum of patient 6 | EMD-45357 |
| Polyclonal immune complex of Fab binding the H3 HA from serum of patient 7 | EMD-45358 |
| Polyclonal immune complex of Fab binding the H1 HA from serum of donor 1 at day 0 | EMD-45310 |
| Polyclonal immune complex of Fab binding the H1 HA from serum of donor 2 at day 0 | EMD-45311 |
| Polyclonal immune complex of Fab binding the H1 HA from serum of donor 3 at day 0 | EMD-45312 |
| Polyclonal immune complex of Fab binding the H1 HA from serum of donor 4 at day 0 | EMD-45313 |
| Polyclonal immune complex of Fab binding the H1 HA from serum of donor 5 at day 0 | EMD-45314 |
| Polyclonal immune complex of Fab binding the H1 HA from serum of donor 6 at day 0 | EMD-45315 |
| Polyclonal immune complex of Fab binding the H1 HA from serum of donor 7 at day 0 | EMD-45316 |
| Polyclonal immune complex of Fab binding the H1 HA from serum of donor 9 at day 0 | EMD-45317 |
| Polyclonal immune complex of Fab binding the H1 HA from serum of donor 10 at day 0 | EMD-45318 |
| Polyclonal immune complex of Fab binding the H3 HA from serum of donor 1 at day 0 | EMD-45319 |
| Polyclonal immune complex of Fab binding the H3 HA from serum of donor 2 at day 0 | EMD-45320 |
| Polyclonal immune complex of Fab binding the H3 HA from serum of donor 3 at day 0 | EMD-45321 |
| Polyclonal immune complex of Fab binding the H3 HA from serum of donor 5 at day 0 | EMD-45322 |
| Polyclonal immune complex of Fab binding the H3 HA from serum of donor 6 at day 0 | EMD-45323 |
| Polyclonal immune complex of Fab binding the H3 HA from serum of donor 7 at day 0 | EMD-45324 |
| Polyclonal immune complex of Fab binding the H3 HA from serum of donor 9 at day 0 | EMD-45325 |
| Polyclonal immune complex of Fab binding the H3 HA from serum of donor 10 at day 0 | EMD-45326 |
| Polyclonal immune complex of Fab binding the H1 HA from serum of donor 1 at day 14 | EMD-45327 |
| Polyclonal immune complex of Fab binding the H1 HA from serum of donor 2 at day 14 | EMD-45328 |
| Polyclonal immune complex of Fab binding the H1 HA from serum of donor 5 at day 14 | EMD-45329 |
| Polyclonal immune complex of Fab binding the H1 HA from serum of donor 6 at day 14 | EMD-45330 |
| Polyclonal immune complex of Fab binding the H1 HA from serum of donor 7 at day 14 | EMD-45331 |
| Polyclonal immune complex of Fab binding the H1 HA from serum of donor 8 at day 14 | EMD-45332 |
| Polyclonal immune complex of Fab binding the H1 HA from serum of donor 9 at day 14 | EMD-45333 |
| Polyclonal immune complex of Fab binding the H1 HA from serum of donor 10 at day 14 | EMD-45334 |
| Polyclonal immune complex of Fab binding the H3 HA from serum of donor 1 at day 14 | EMD-45335 |
| Polyclonal immune complex of Fab binding the H3 HA from serum of donor 2 at day 14 | EMD-45336 |
| Polyclonal immune complex of Fab binding the H3 HA from serum of donor 3 at day 14 | EMD-45337 |
| Polyclonal immune complex of Fab binding the H3 HA from serum of donor 4 at day 14 | EMD-45338 |
| Polyclonal immune complex of Fab binding the H3 HA from serum of donor 5 at day 14 | EMD-45339 |
| Polyclonal immune complex of Fab binding the H3 HA from serum of donor 6 at day 14 | EMD-45340 |
| Polyclonal immune complex of Fab binding the H3 HA from serum of donor 7 at day 14 | EMD-45341 |
| Polyclonal immune complex of Fab binding the H3 HA from serum of donor 8 at day 14 | EMD-45342 |
| Polyclonal immune complex of Fab binding the H3 HA from serum of donor 9 at day 14 | EMD-45343 |
| Polyclonal immune complex of Fab binding the H3 HA from serum of donor 10 at day 14 | EMD-45344 |
